# Transcriptomic analysis in the striatum reveals the involvement of Nurr1 in the social behavior of prenatally valproic acid-exposed male mice

**DOI:** 10.1038/s41398-022-02056-z

**Published:** 2022-08-09

**Authors:** Hyunju Kim, Ran-Sook Woo, Eun-Jeong Yang, Han-Byeol Kim, Eun hwa Jo, Sangjoon Lee, Hehin Im, Seonghan Kim, Hye-Sun Kim

**Affiliations:** 1 Department of Pharmacology and Biomedical Sciences, College of Medicine, Seoul National University, 103 Daehakro, Jongro-gu, Seoul, Republic of Korea; 2 Neuroscience Research Center, College of Medicine, Seoul National University, 103 Daehakro, Jongro-gu, Seoul, Republic of Korea; 3 Department of Anatomy and Neuroscience, College of Medicine, Eulji University, Daejeon, 34824 Republic of Korea; 4 Convergence Research Center for Diagnosis, Treatment and Care System of Dementia, Korea Institute of Science and Technology (KIST), Seoul, Republic of Korea; 5 Brain Science Institute, Korea Institute of Science and Technology (KIST), Seoul, Republic of Korea; 6 Division of Bio-Medical Science & Technology, KIST School, Korea University of Science and Technology, Seoul, Korea; 7 Department of Anatomy, College of Medicine, Inje University Bokji-ro 75, Busanjin-gu, Busan, 47392 Korea; 8 Seoul National University College of Medicine, Bundang Hospital, 13620, Seongnam, Republic of Korea

**Keywords:** Molecular neuroscience, Autism spectrum disorders

## Abstract

Autism spectrum disorder (ASD) is a neurodevelopmental disorder that exhibits neurobehavioral deficits characterized by abnormalities in social interactions, deficits in communication as well as restricted interests, and repetitive behaviors. The basal ganglia is one of the brain regions implicated as dysfunctional in ASD. In particular, the defects in corticostriatal function have been reported to be involved in the pathogenesis of ASD. Surface deformation of the striatum in the brains of patients with ASD and their correlation with behavioral symptoms was reported in magnetic resonance imaging (MRI) studies. We demonstrated that prenatal valproic acid (VPA) exposure induced synaptic and molecular changes and decreased neuronal activity in the striatum. Using RNA sequencing (RNA-Seq), we analyzed transcriptome alterations in striatal tissues from 10-week-old prenatally VPA-exposed BALB/c male mice. Among the upregulated genes, Nurr1 was significantly upregulated in striatal tissues from prenatally VPA-exposed mice. Viral knockdown of Nurr1 by shRNA significantly rescued the reduction in dendritic spine density and the number of mature dendritic spines in the striatum and markedly improved social deficits in prenatally VPA-exposed mice. In addition, treatment with amodiaquine, which is a known ligand for Nurr1, mimicked the social deficits and synaptic abnormalities in saline-exposed mice as observed in prenatally VPA-exposed mice. Furthermore, PatDp+/− mice, a commonly used ASD genetic mouse model, also showed increased levels of Nurr1 in the striatum. Taken together, these results suggest that the increase in Nurr1 expression in the striatum is a mechanism related to the changes in synaptic deficits and behavioral phenotypes of the VPA-induced ASD mouse model.

## Introduction

The striatum is the major input structure for the basal ganglia, and it integrates excitatory inputs from various brain regions, including the cortex and thalamus, to control diverse functions [1, 2]. Although the striatum controls motor movement, recent studies confirmed that this region was related to goal-directed actions, habitual actions, and motivation [3, 4]. Magnetic resonance imaging (MRI) studies implicated activation of the striatum in response to social behaviors, including social rewards [5, 6], and a positive correlation with reciprocal social interaction and communicative skills in the bilateral medial caudate head [7]. The striatum consists of the dorsomedial striatum (DMS), dorsolateral striatum (DLS), and ventral striatum (VS). Importantly, the DMS receives afferent projections from the associative cortices, thalamus, hippocampus, and amygdala [8]. The synaptic connections observed in different brain regions such as the corticostriatal and thalamostriatal circuits have been shown to be critical for social deficits in various ASD mouse models [9–11]. Therefore, the synaptic plasticity in DMS could be important in sociability.

Medium spiny neurons, which account for ~90–95% of all neurons in the striatum and use γ-aminobutyric acid (GABA) as a neurotransmitter, have two subtypes that are differentiated by their enrichment of dopamine receptor 1 (D1) or D2. Several ASD mouse model studies reported synaptic defects in D1 or D2-expressing medium spiny neurons in the striatum. The numbers of D2 medium spiny neurons were increased in the striatum of 16p11^+/−^ mice [12], and the repetitive behaviors of Shank3B-KO mice were rescued by enhancing D2 medium spiny neuron activity [13]. Notably, neuroligin-3 mutations caused a proxy for acquired repetitive behaviors in mice via synaptic impairment in D1 medium spiny neurons of the VS [14].

Genetic, epigenetic, and environmental factors are related to the induction of ASD. The investigation on ASD postmortem brains using RNA-Seq provided evidence for the enrichment of immune-related genes and the downregulation of neuronal and synaptic genes in autism [15, 16]. The induced pluripotent stem cell-derived neurons from patients with idiopathic ASD showed dysregulation of genes involved in neuronal differentiation, axon guidance, cell migration, DNA and RNA metabolism, and neural region patterning [17]. A comparative gene expression profile of the hippocampus from two genetic mouse models of ASD (BTBR and En2-/- mice) exhibited represented a specific enrichment profile in neuronal and glial genes and genes associated with ASD comorbidities [18]. Overall, these studies suggest a convergent pathway between ASD patients and mouse models.

Our study used striatal tissues obtained from prenatally VPA-exposed mice to investigate transcriptomic dysregulation and its role in the pathophysiology of ASD. Clinical studies have demonstrated that the use of VPA during pregnancy increased the risks of intellectual disability and ASD behavioral symptoms in children [19, 20]. In accordance with clinical evidence, mice prenatally exposed to VPA show behavioral deficits in social interaction, locomotor activity, and repetitive behaviors [21]. In the present study, we performed a battery of behavioral tests for core behavioral symptoms, including developmental delay, impaired social interactions, and repetitive and stereotyped behaviors. Prenatally VPA-exposed mice exhibited delayed development in the self-righting test, decreased social interaction in the maternal scent preference and three-chamber tests, and accelerated repetitive motor learning in the rotarod test. In addition to the behavioral changes, prenatal VPA exposure induces cellular and molecular abnormalities, including neuron mislocalization [22, 23] and changes in the expression of synaptic proteins [24, 25]. Among the differentially expressed genes (DEGs) in the striatum of prenatally VPA-exposed mice, Nurr1 was significantly upregulated at the transcriptional and protein levels in the striatum of prenatally VPA-exposed mice.

Nurr1 is an orphan receptor that plays an essential role in dopaminergic neuron development, maintenance, differentiation, and survival [26]. Nurr1 regulates multiple necessary proteins and activates tyrosine-protein kinase receptor Ret to facilitate dopaminergic neuron growth and survival [27, 28]. Nurr1 dysregulation was reported in some neurodevelopmental diseases. Genome and exome sequencing data comparisons from ASD families showed a frameshift variant in Nurr1 [29].

In this study, we showed for the first time that the expression of Nurr1 was increased in the striatum of prenatally VPA-exposed mice, and Nurr1 knockdown by shRNA rescued social deficits observed in prenatally VPA-exposed mice. Also, Nurr1 activation by its ligand, AQ, in saline-exposed mice induced a reduction in dendritic spine density in the striatum and impaired social interaction as shown in VPA-exposed mice. In addition, we found that Nurr1 expression was upregulated in the striatum of a commonly used ASD genetic mouse model, PatDp^+/-^ mice which carry a 6.3 Mb paternal duplication homologous to the human 15q11-q13 locus. Taken together, the Nurr1 may be used as a therapeutic target for ASD.

## Materials and methods

### Animals

BALB/c mice were purchased from Koatech (Pyeong-Taek, Korea) and mated. On TP 12.5, pregnant BALB/c mice were subcutaneously injected with a single dose of VPA (600 mg/kg in saline) or vehicle saline (SAL). Only male pups were included in the present study. All animal experiments were approved by the Animal Care Committee of Seoul National University, Seoul, Republic of Korea (Approval number: SNU-190426-10-1). Animals were housed at a temperature of 24 ± 1°C with a 12/12 h light/dark cycle with free access to food and water.

### RNA-Seq

RNA library preparation, cluster generation, and sequencing were performed by TheragenEtex BiO Institute (Suwon, Korea). The cDNAs were subjected to end-repair, poly A addition, and connected with sequencing adapters using the TruSeq RNA Library prep Kit (Illumina, CA, USA). The suitable fragments automatically purified by BluePippin 2% agarose gel cassette (Sage Science, MA, USA) were selected as templates for PCR amplification. Subsequently, the library was sequenced using an Illumina HiSeq2500 sequencer (Illumina, CA, USA). For differential expression analysis, gene-level count data were generated using the HTSeq-count v0.5.4p3 tool with the option “-m intersection-nonempty” and -r option considering paired-end sequence. Based on the calculated read count data, DEGs were identified using the R package called TCC. The TCC package applies robust normalization strategies to compare tag count data. Normalization factors were calculated using the iterative DEGES/edgeR method. The *Q*-value was calculated based on the *p* value using the *p*.adjust function of R package with default parameter settings. DEGs were identified based on *q*-value threshold <0.05.

### Bioinformatic analysis

The gene ontology analyses of differentially expressed genes were performed using DAVID software (version 6.8). Differentially expressed genes were also analyzed for phenotypes using mouse genome informatics mammalian phenotype analysis in Enrichr (http://amp.pharm.mssm.edu/Enrichr/). *p*-value 0.05 was used as a cutoff. For pathway analyses, pathways with more than 7 members were included.

### Amodiaquine (AQ) treatment

Six-wee-old mice were intraperitoneally injected with amodiaquine (AQ, Sigma-Aldrich, MO, USA, 20 mg/kg), twice per day at 12 h intervals, for 2 weeks. Mice underwent behavioral testing 1 week after the final injection. Brain samples were harvested 2–3 weeks after the final injection. Brain samples were harvested 2–3 weeks after the final injection.

### Stereotaxic injection of lentivirus

Nurr1 and its control shRNA lentiviruses were purchased from Sirion Biotech (Martinsried, Germany). Three-week-old mice received bilateral stereotaxic injections of virus (1.5 μl per side) into the dorsal striatum (coordinates: AP + 0.3, ML ± 1.9, DV −3.25 mm) at rates of 0.15 μl/min at each site (Kopf instruments, CA, USA). Mice underwent behavioral testing 5 weeks after the lentivirus injection. Brain samples were harvested 7 weeks after the lentivirus injection.

### Golgi staining

Golgi staining was performed using the FD Rapid GolgiStain Kit (FD Neurotechnologies, MD, USA) according to the manufacturer’s instructions. To assess spine density and morphological phenotype, 8–10 cells of each slice were randomly selected. 2–3 dendrites per neuron were analyzed. Stacks of 512 × 512 pixel 3-D images with an interval of 1 μm were then taken for each cell to include all visible dendritic branches in the Zen software. After 3D neuronal reconstruction, the secondary and tertiary dendrite spines were measured, wherein the distance to the soma varied from 20–80 µm.

### Quantitative reverse transcription polymerase chain reaction (qPCR)

Extracted total RNA was converted to cDNA using AccuPower RocketScript RT PreMix (Bioneer, Daejeon, Korea). qPCR was performed using a CFX96 (Bio-Rad, CA, USA). Results are presented as △△Ct-values normalized to the 18S rRNA. Primers were designed using NCBI primer blast software (http://www.ncbi.nlm.nih.gov/tools/primer-blast/).

### Western blot

Protein was quantified using a bicinchoninic acid assay kit (Thermo Fisher Scientific, IL, USA). 20–50 μg of proteins were resolved on a 10% SDS-PAGE gel or tris-tricine gel and transferred to nitrocellulose or polyvinylidene fluoride membrane, followed by blocking with 5% skim milk. Information on the antibodies used is described in ‘Supplementary Methods’.

### Behavioral assays

A behavior test was performed between 12:00 pm and 6:00 pm. Social interaction was assayed using the three-chamber test. The apparatus was constructed of a Plexiglas box (60 × 45 × 22 cm) partitioned into three chambers with retractable doorways. Openings between the compartments allowed the animals to access all three chambers. The test was performed in three phases. During the first phase (habituation), a test mouse was placed in the box for 5 min. After this phase, two cups with small holes allowing olfactory contact between mice were placed in two peripheral chambers. An age- and sex-matched mouse (stranger in the sociability test, familiar in the social novelty preference test) was placed under the cup. The second cup remained empty. A test mouse was placed in the center chamber and allowed to freely explore for 10 min (sociability test). Immediately after the second phase, the entrances were blocked. A new unfamiliar mouse (novel) of the same age and sex as the test mouse was placed under the second cup, and the tested animal remained in a box for an additional 10 min (social novelty preference test). The order in which mouse was used in behavior tests was randomized between litters. The analysis was performed by an operator blinded to the groups. The procedures of self-righting, maternal scent preference, rotarod, and open field tests are described in ‘Supplementary Methods’.

### Immunofluorescence and image analysis

The brains of 9–10-week-old VPA- or SAL mice were then removed and post-fixed in 4% paraformaldehyde at 4 °C for 24 h before they were transferred to 30% sucrose-PBS 0.1 M, pH 7.3 solution at 4 °C. Afterward, the brains were sectioned into 30 μm-thick coronal sections using a cryostat (Thermo Fisher Scientific, IL, USA), and three slices per mouse were used in all IF analyses (*n* = 3–4 mice/staining). Sections were then incubated in blocking buffer containing primary antibodies diluted in blocking buffer at 4 °C overnight. Next, sections were incubated with the secondary antibodies in PBS for 2–3 h at room temperature. Finally, sections were stained with Topro3 (diluted 1:1000; Thermo Fisher Scientific, IL, USA) or DAPI (1:1000, D1306, Thermo Fisher) in PBS. The images were acquired on an LSM510 confocal microscope (Zeiss, Oberkochen, Germany) using a Plan-Neofluar 40x/0.90 N.A. with a water immersion objective or on a Nikon A1 confocal microscope (Nikon, Melville, NY, USA) with a Plan fluor 20× lens (0.75 numerical aperture). For quantification, 2**–**3 striatal regions were randomly selected for confocal imaging, wherein the intensity of each region was analyzed. Information on the antibodies used is described in ‘Supplementary Methods’.

### Multi-electrode array (MEA)

For solutions, standard artificial cerebrospinal fluid (ACSF): 125 mM NaCl, 2.5 mM KCl, 1.25 mM NaH_2_PO_4_, 1.9 mM MgSO_4_, 20 mM Glucose, 25 mM NaHCO_3_, 2 mM CaCl_2_. Dissection ACSF: standard ACSF without CaCl_2_. Brains were dissected out and immediately immersed in ice-cold dissection ACSF. After brains were glued onto the vibratome tray, slices were cut and bubbled with carbogen (95% O_2_ and 5% CO_2_) during slicing. The slices were transferred to carbogen-bubbled warm ACSF (35 °C) and were allowed to recover for at least 1 h before placement on Axion BioSystems Maestro MEA. Neuronal activity of firings induced by electrical stimulations were detected on the plate and the number of firings from each stimulation current with firing heat-maps were analyzed via software AxIS Navigator™ (2.0.4.).

### Statistical analysis

Data are expressed as means ± SEM values and were analyzed with the SPSS 23 software (IBM, Chicago, IL, USA) using the Kruskal–Wallis test, one-way-ANOVA with LSD post hoc analysis, two-way-ANOVA with LSD post hoc analysis, or repeated measures (RM)-ANOVA with Bonferroni post hoc analysis. The results were considered to be statistically significant if *p* < 0.05. n means a number of mice analyzed unless stated otherwise.

## Results

### Abnormalities in the density and morphology of dendritic spines in the striatum of prenatally VPA-exposed 10-week-old mice

**Fig. 1:**
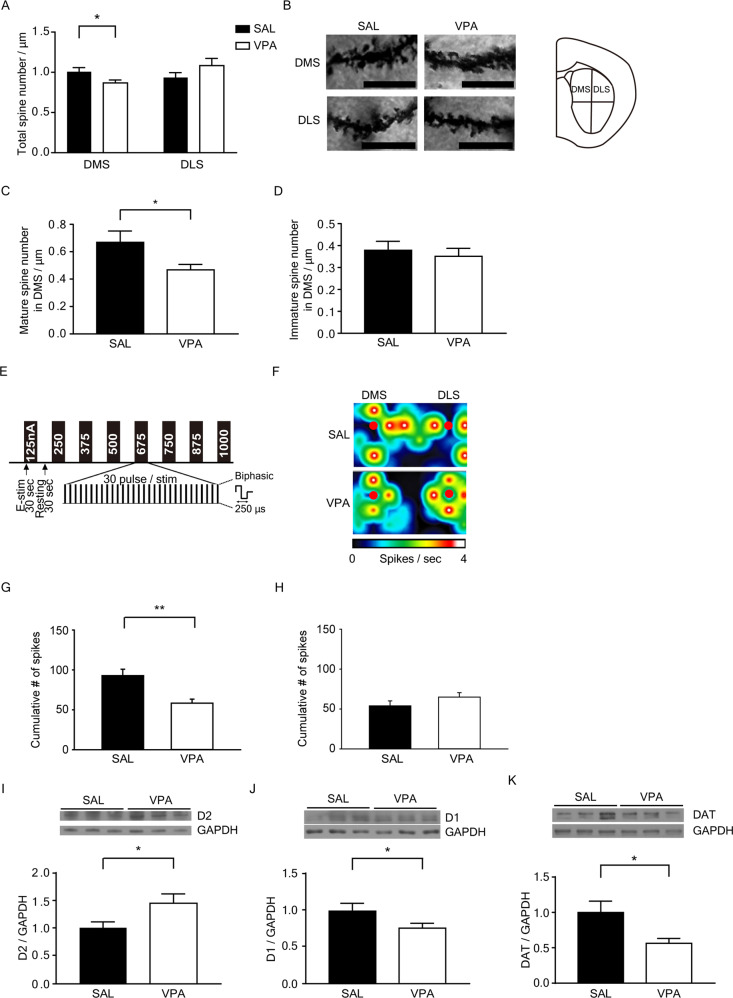
Abnormalities in dendritic spine density and inputs in the striatum of prenatally VPA-exposed 10-week-old mice. **A** Quantification of total dendritic spine density (F(1, 60) = 6.103, *p* = 0.016, interaction; F(1, 60) = 0.027, *p* = 0.870, group; F(1, 60) = 1.88, *p* = 0.175, region, two-way ANOVA, SAL DMS, *n* = 21; VPA DMS, *n* = 20; SAL DLS, *n* = 16; VPA DLS, *n* = 10). **B** Representative images of Golgi-stained neurons in the DMS and DLS from SAL and VPA mice. Scale bars: 10 μm. **C**, **D** Quantification of mature and immature dendritic spines/ μm (SAL, *n* = 7; VPA, *n* = 9). *n* means the number of neurons analyzed. **E** Diagram showing protocol summary of MEA experiment showing the stepwise increasing stimulation procedures **F** Representative heat-map image on the MEA probes (red dots: stimulation positions) **G** Bar graphs showing the cumulative number of firings from all stimulation currents in DMS (mean ± SEM, SAL, *n* = 20; VPA, *n* = 16 brain slices from 6 mice, respectively). **H** Bar graphs showing the cumulative number of firings from all stimulation currents in DLS (mean ± SEM, SAL, *n* = 14; VPA, *n* = 14 brain slices from six mice, respectively) **I**–**K** Representative blots for D2, D1 and DAT and densitometric analysis of striatal D2 receptor (SAL, *n* = 11; VPA, *n* = 11), D1 receptor (SAL, *n* = 20; VPA, *n* = 20), and DAT levels (SAL, *n* = 11; VPA, *n* = 11). Data are presented as the means ± SEM. *p < 0.05, **p < 0.01 compared to SAL mice, unpaired *t*-test.

Morphological abnormalities of the striatum are one of the most consistent abnormalities reported in ASD [30, 31]. To analyze the effects of VPA on dendritic structure in the striatum, Golgi staining was performed on the brain tissues of 10-week-old mice. Notably, VPA mice had a significantly reduced spine density compared to SAL mice in the DMS (*p* = 0.036), but not in the DLS (Fig. 1A). Figure 1B shows the representative images of Golgi-stained neurons in the DMS and DLS. We investigated the morphology of the dendritic spines. Dendritic spines are classified by the length and width of the spine head as filopodia, thin, stubby, mushroom, and branch types [32]. The filopodia and thin types were categorized as immature spines, and the stubby, mushroom, and branch types were categorized as mature spines. The number of mature spines was significantly decreased in DMS (*p* = 0.033) (Fig. 1C). In contrast, the number of immature spines in the DMS of VPA mice was not different than SAL mice (Fig. 1D).

To test the neuronal activity of the striatum, brain slices of the striatum regions were analyzed on the MEA with 64 electrodes in each well. The stimulus protocol was shown in Fig. 1E. DMS from VPA mice showed significantly reduced neuronal activity (Fig. 1G, *p* = 0.016), while DLS did not (Fig. 1H, *p* = 0.393). The number of firings of striatal neurons following stepwise increasing stimulation currents showed a significantly reduced pattern of neuronal activities in all stimulation currents in the DMS, but only in the lowest and high stimulation currents in the DLS of the VPA mice (Supplementary Fig. 1A, B). Representative heat-map images of neuronal activity in the MEA probes showed a decreased firing pattern in the DMS of VPA mice (Fig. 1F).

The principal neuron type in the striatum is medium spiny neurons expressing dopamine receptors. We further analyzed the differential expression of D1 or D2 to examine whether prenatal VPA exposure was involved in the dysregulation of dopamine receptor expression. The expression level of D2, which indicates the population of D2 medium spiny neurons, was increased in the striatal tissues of prenatally VPA-exposed mice (*p* = 0.038) (Fig. 1I). D1 expression level was decreased (*p* = 0.032) (Fig. 1J). Full western blot images for D1 and D2 are shown in Supplementary Fig. 8.

The main inputs to the striatum are glutamatergic signals from the cerebral cortex and thalamus and dopaminergic signals from the substantia nigra/ventral tegmental area (SN/VTA). Vesicular glutamate transporter 1 (VGLUT1) and VGLUT2 are localized to excitatory nerve terminals projecting from cortical and thalamic areas, respectively, in the striatum [33]. The expression of VGLUT1, which represents corticostriatal terminals in the striatum, was decreased in the striatum of VPA mice (*p* = 0.017) (Supplementary Fig. 1C, D). The expression of DAT, which is a marker for nigrostriatal dopaminergic nerve terminals, was decreased in the striatum of VPA mice (*p* = 0.021) (Fig. 1K). The dysfunction of glutamatergic and dopaminergic terminals in the striatum may underlie the reduction in spine density in the DMS of VPA mice.

### Striatal transcriptome analysis of prenatally VPA-exposed 10-week-old mice

**Fig. 2:**
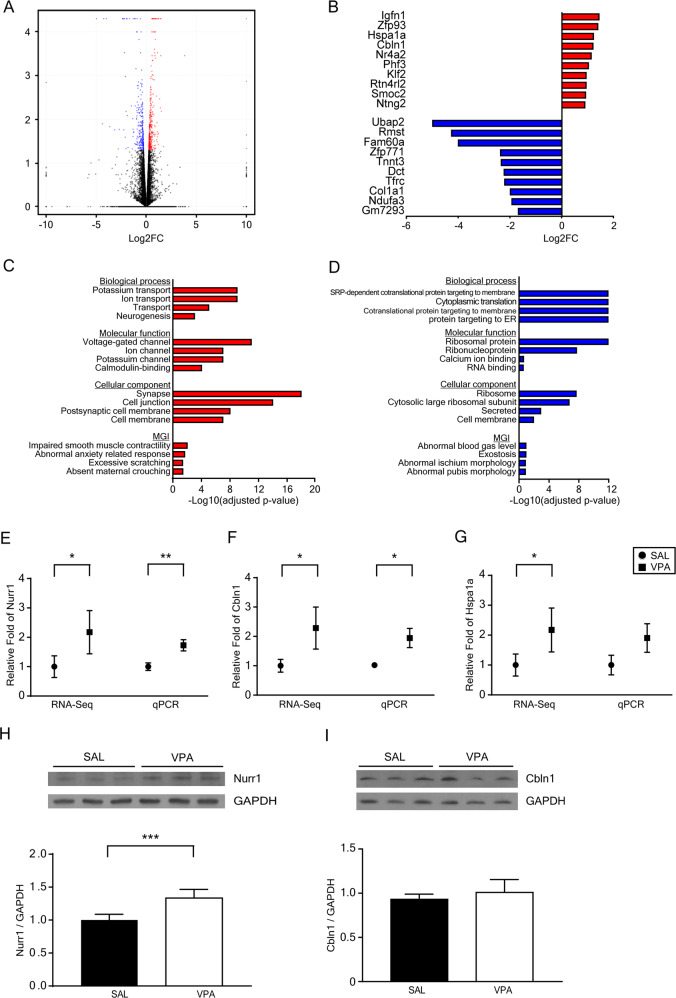
Striatal transcriptome analysis of prenatally VPA-exposed 10-week-old mice. **A** Volcano plot depicting 348 significantly upregulated and 258 significantly downregulated genes (log2-fold change). **B** List of the top 10 upregulated and downregulated differentially expressed genes (based on the fold change) from the RNA-Seq analysis. **C**, **D** GO and mouse genome informatics mammalian phenotype analysis of the upregulated genes and downregulated genes. **E**–**G** mRNA expression of Nurr1 (SAL, *n* = 9; VPA, *n* = 9), Cbln1 (SAL, *n* = 11; VPA, *n* = 11), and Hspa1a (SAL, *n* = 7; VPA, *n* = 8) in the prenatally VPA-exposed striatum determined by RNA-Seq and qPCR. compared to SAL mice. **H**, **I** Representative blots for Nurr1 and Cbln 1 and densitometric analysis of protein levels of Nurr1 (SAL, *n* = 12; VPA, *n* = 12) and Cbln1 (SAL, *n* = 18; VPA, *n* = 18) in the striatum of VPA mice *p < 0.05, **p < 0.01, ***p < 0.001 compared to SAL mice, unpaired *t*-test, or Mann–Whitney test.

To identify transcriptome profiles in the striatum of VPA or SAL mice, RNA-Seq with striatal mRNA from 10-week-old mice was performed. A total of 348 genes were upregulated while 258 genes were downregulated in the striatum of the VPA mice compared to SAL mice (Fig. 2A). The top 10 differentially expressed genes are listed in Fig. 2B.

We analyzed the gene ontology (GO) of genes that had a *p*-value < 0.05 to identify molecular and physiological signatures. Notably, upregulated genes were related to “ion transport” and “neurogenesis” in biological process, and “ion channel (voltage-gated and potassium)” and “calmodulin binding” in molecular function which are important to the excitability of neurons. Analysis of GO terms by cellular component showed that upregulated genes were expressed mostly in neuron-specific parts, such as “synapse”, and “postsynaptic cell membrane”. Upregulated genes for the enrichment of mouse phenotype terms were also tested. Two ASD-related phenotypes, “abnormal anxiety-related response” and “excessive scratching” were enriched. An additional GO term analysis of downregulated genes revealed that “protein targeting to endoplasmic reticulum or membrane”, and “cytoplasmic translation” in biological processes, “ribosomal protein”, “calcium ion binding”, and “RNA binding” in molecular function, and “ribosome”, “secreted”, and “cell membrane” in the cellular component were related. Downregulated genes showed relevance to the bone development-related mouse phenotype (“exostosis”, “abnormal ischium morphology”, and “abnormal pubis morphology”) (Fig. 2C, D).

Because the upregulated gene sets exhibited more relevance to neuronal function and ASD symptoms than the downregulated gene sets, we specifically focused on the upregulated genes. To verify these genes, the top 10 upregulated genes were analyzed using qPCR with striatal mRNA of 10-week-old mice. Nurr1 (*p* = 0.007) and Cbln1 mRNA (*p* = 0.014) expression levels were increased using qPCR (Fig. 2E, F). Hspa1a mRNA was increased using RNA-Seq, but showed only a tendency to increase following qPCR (Fig. 2G).

To demonstrate the changes in protein expression, we performed a Western blot analysis on the two genes that were increased using RNA-Seq and qPCR. We identified that only the Nurr1 protein level was significantly increased in the striatum of 10-week-old VPA mice (*p* = 4.55 × 10^−5^) (Fig. 2H, I).

### Lentiviral Nurr1 knockdown in the striatum rescues autism-like social deficits and molecular/synaptic alterations in prenatally VPA-exposed 10-week-old mice

First, we investigated cell type-specific expression of Nurr1 in the brain. It was found that 84.51 ± 2.33% of Nurr1-expressing cells were neurons and 8.02 ± 1.08% were microglia (*p* = 1.43 × 10^−14^) (Supplementary Fig. 2A, B). Based on this result, we designed a vector expressing shRNA under the Syn promoter. Astrocytes only reside in or around the striosomes, and these cells did not colocalize with Nurr1 positive cells in the striatum (data not shown).

**Fig. 3:**
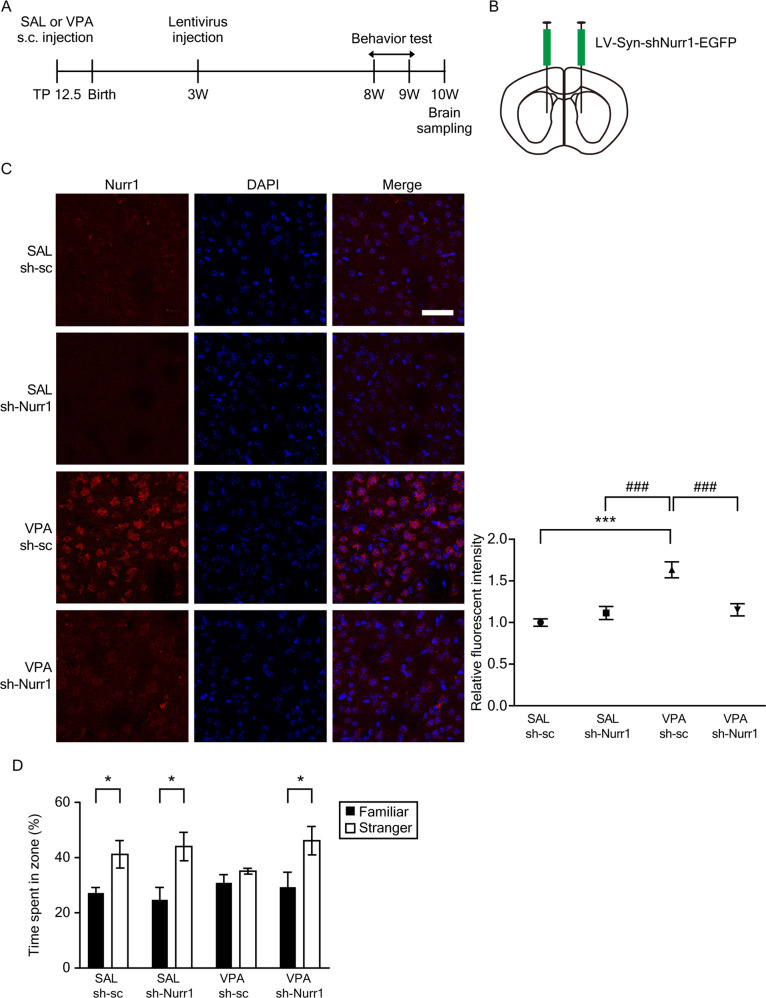
Lentiviral Nurr1 knockdown in the striatum rescued autism-like social deficits in prenatally VPA-exposed 10-week-old mice. **A**, **B** Schematic illustrations displaying the experimental paradigms of behavior tests (**A**) and lentiviral injection (**B**). **C** Representative figures of immunohistochemical experiments and the quantification of Nurr1^+^ immunofluorescence intensity (SAL Veh, *n* = 5; SAL sh-Nurr1, *n* = 7; VPA Veh, *n* = 5; VPA sh-Nurr1, *n* = 7). ***p < 0.001 compared to SAL sh-sc mice, ^###^*p* < 0.001 compared to VPA sh-sc mice, one-way ANOVA. **D** The interaction time with familiar and novel mice (*F*(3, 60) = 1.329, *p* = 0.277, interaction; *F*(3, 60) = 0.056, *p* = 0.946, group; *F*(1, 60) = 11.509, *p* = 0.002, familiar vs. novel, two-way ANOVA, SAL sh-sc, *n* = 8; SAL sh-Nurr1, *n* = 8; VPA sh-sc, *n* = 9; VPA sh-Nurr1, *n* = 9). *p < 0.05 compared to time in zone with familiar mouse, unpaired *t*-test.

Then, to examine the therapeutic potential of Nurr1 knockdown, we stereotaxically injected lentiviruses expressing shRNA targeting Nurr1 into the dorsal striatum. Schematic illustrations regarding the experimental procedures and the stereotaxic injection into the dorsal striatum were shown in Figs. 3A, B, respectively. The viral spread after stereotaxic injection as assessed with GFP staining was confirmed only in the dorsal striatum (Supplementary Fig. 3A, B). The knockdown of Nurr1 was confirmed in virus-infected striatum tissue from prenatally VPA-exposed mice (*p* = 4.75 × 10^−8^, VPA sh-Nurr1 mice compared to VPA sh-sc mice) (Fig. 3C).

Next, we assessed the resulting behavioral consequences. In SAL mice, the effect of shRNA was insignificant because the basal intensity was very low in the mice. To investigate the effect of Nurr1 knockdown on sociability and social novelty preference, we performed a three-chamber test. In the sociability test, SAL sh-sc mice, SAL sh-Nurr1 mice, and VPA sh-Nurr1 mice spent more time in a chamber containing an age- and sex-matched mouse than in a chamber with an object (Supplementary Fig. 3C, SAL sh-sc, *p* = 0.039; SAL sh-Nurr1, *p* = 0.024; VPA sh-sc, *p* = 0.792; VPA sh-Nurr1; *p* = 0.024). In the social novelty preference test, VPA sh-Nurr1 mice exhibited a significantly increased social interaction time, compared to VPA sh-sc mice in three-chamber test (Fig. 3D, SAL sh-sc, *p* = 0.020; SAL sh-Nurr1, *p* = 0.014; VPA sh-sc, *p* = 0.205; VPA sh-Nurr1; *p* = 0.039). Taken together, these results indicated that Nurr1 knockdown rescued behavioral deficits in sociability and social novelty preference in VPA mice.

**Fig. 4:**
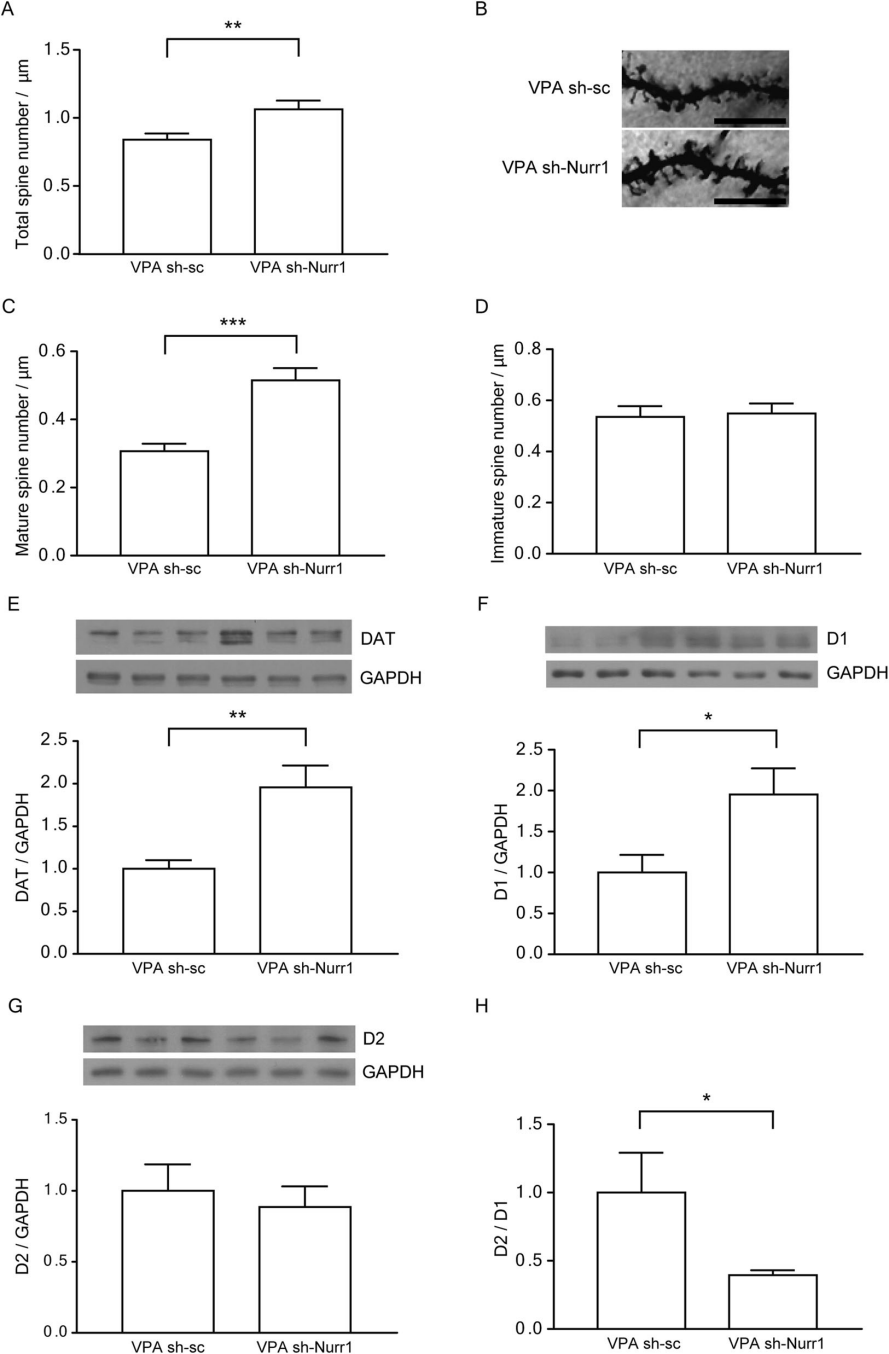
Lentiviral Nurr1 knockdown in the striatum rescued the abnormalities in dendritic spine density and inputs in the striatum of prenatally VPA-exposed 10-week-old mice. **A** Quantification of total dendritic spine numbers/ μm in VPA sh-sc and VPA sh-Nurr1 mice. **B** Representative images of Golgi-stained neurons in the DMS from VPA sh-sc and VPA sh-Nurr1 mice. Scale bars represent 10 μm. **C**, **D** Quantification of mature (**C**) and immature dendritic spine density (**D**) (VPA sh-sc, *n* = 15; VPA sh-Nurr1, *n* = 11). *n* means the number of neurons analyzed. **p < 0.01, ***p < 0.001 compared to VPA sh-sc mice, unpaired *t*-test. **E**–**G** Representative blots for DAT, D1 and D2 and the densitometric analysis of protein levels of DAT (VPA sh-sc, *n* = 7; VPA sh-Nurr1, *n* = 8), D1 (VPA sh-sc, *n* = 6; VPA sh-Nurr1, *n* = 8), and D2 (VPA sh-sc, *n* = 7; VPA sh-Nurr1, *n* = 8). **H** The relative expression of D2 compared to D1 (VPA sh-sc, *n* = 5; VPA sh-Nurr1, *n* = 8). *p < 0.05, **p < 0.01 compared to VPA sh-sc mice, unpaired *t*-test.

Next, to investigate the effects of Nurr1 knockdown on dendritic structure in the DMS, we analyzed spine density, and found that total (Fig. 4A, B, *p* = 0.007) and mature (Fig. 4C, *p* = 0.00003) spine densities were significantly increased in the DMS of VPA sh-Nurr1 mice. In contrast, the immature spine density in the DMS of VPA sh-Nurr1 mice was not different from that of VPA sh-sc mice (Fig. 4D). We also performed a Western blot analysis. DAT (*p* = 0.009) and D1 (*p* = 0.04) expression levels were significantly increased in the striatum of VPA sh-Nurr1 mice compared to VPA sh-sc mice (Fig. 4E, F). D2 expression level was not significantly different (Fig. 4G), while the relative expression level of D2 compared to D1 was decreased (*p* = 0.023) (Fig. 4H).

### Effects of AQ, a Nurr1 agonist, on cell viabilities in primary striatal neuron cultures

Next, we tested whether Nurr1 activation in primary striatal neuron cultures induced the molecular changes that were observed in the striatum of VPA mice. To identify an optimal concentration that did not induce cytotoxicity, the cell viabilities were evaluated after a 24 h treatment with various concentrations of AQ (10 nM–10 μM) using an MTT assay. Treatment with AQ at concentrations >500 nM was significantly cytotoxic to primary striatal neurons (VPA; *p* = 0.003) (Supplementary Fig. 4A, B). Based on these results, primary striatal neuron cultures from SAL or VPA mice were treated with 100 nM AQ in subsequent experiments. AQ (100 nM) increased D2 expression levels in primary striatal neuron cultures of SAL mice but not VPA mice (*p* = 0.043) (Supplementary Fig. 4C, D).

### Administration of AQ induces ASD-like behaviors in 10-week-old mice

**Fig. 5:**
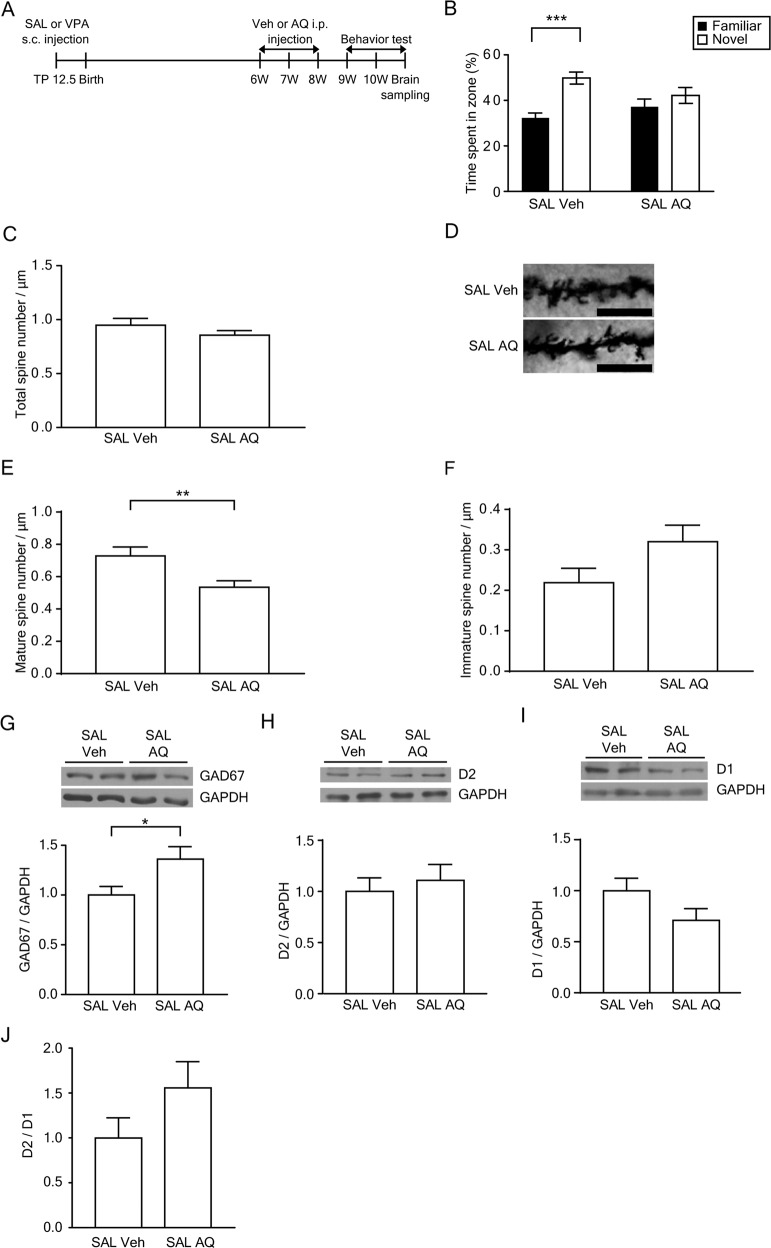
Administration of AQ induced deficits in social interaction in 10-week-old mice. **A** The experimental scheme. **B** The interaction time with familiar and novel mice (*F*(1, 40) = 4.506, *p* = 0.040, interaction; *F*(1, 40) = 0.216, *p* = 0.645, group; *F*(1, 40) = 14.469, *p* = 0.0005, familiar vs. novel, two-way ANOVA, SAL Veh, *n* = 11; SAL AQ, *n* = 11). ***p < 0.001 compared to time in zone with familiar mouse, unpaired *t*-test. **C** Quantification of total dendritic spine density (SAL Veh, *n* = 12; SAL AQ, *n* = 18). **D** Representative images of Golgi-stained neurons in the DMS from SAL Veh and SAL AQ mice. Scale bars represent 10 μm. **E**, **F** Quantification of mature (**E**) and immature (**F**) dendritic spine density (SAL Veh, *n* = 12; SAL AQ, *n* = 18). **p < 0.01 com*p*ared to SAL Veh mice, unpaired *t*-test. *n* means the number of neurons analyzed. **G** Representative blots for GAD67 and densitometric analysis of GAD67, a marker of GABAergic neurons (SAL Veh, *n* = 8; SAL AQ, *n* = 8). **H**, **I** Representative blots for D2 and D1 and the densitometric analysis of protein levels of D2 (SAL Veh, *n* = 10; SAL AQ, *n* = 8) and D1 (SAL Veh, *n* = 13; SAL AQ, *n* = 12), unpaired *t*-test. **J** The relative expression of D2 compared to D1 (SAL Veh, *n* = 11; SAL AQ, *n* = 8). *p < 0.05 compared to SAL Veh mice, Mann–Whitney test.

To characterize the effect of Nurr1 activation on behavior, SAL mice were injected with 20 mg/kg AQ for 14 days and subjected to a battery of behavioral tests (Fig. 5A). The body and brain weights of the SAL AQ mice were similar to the SAL veh mice (Supplementary Fig. 5B, C). To examine the role of AQ in ASD-like behaviors, social behavioral abnormalities were investigated using a three-chamber test. In the sociability test, SAL AQ mice showed no preference for a stranger mouse over an object (Supplementary Fig. 5A, SAL Veh; *p* = 0.032, SAL AQ; *p* = 0.172). In the social novelty preference test, SAL AQ mice exhibited no difference in the time spent in the familiar zone and novel zone (Fig. 5B, SAL Veh; *p* = 0.00005, SAL AQ; *p* = 0.317). Rotarod test was performed to evaluate motor coordination and accelerated motor learning. The result of AQ-injected SAL mice in trials 1, 2, and 3 indicated that this mice group displayed a significantly increased motor learning rate than the vehicle-injected SAL mice (T1; *p* = 0.04, T2; *p* = 0.014, T3; *p* = 0.028) (Supplementary Fig. 5D). During the open field test, the AQ-injected SAL mice showed comparable motor function with the vehicle group (Supplementary Fig. 5E). Collectively, these results indicate that Nurr1 affects sociability, social novelty preference, and repetitive motor learning.

### Synaptic and molecular changes in the striatum of AQ-injected 10-week-old mice

To elucidate the underlying functional mechanisms responsible for the decreased social activity in AQ-injected SAL mice, dendritic spine density was measured in the DMS. The density of mature spines was decreased in the DMS of AQ-injected SAL mice (*p* = 0.007) (Fig. 5E). The densities of total spines and immature spines were not significantly different (Fig. 5C, F). Figure 5D shows representative images of Golgi-stained neurons in the DMS.

To determine the molecular changes related to medium spiny neurons, the expression of GAD67, D2, and D1 was examined in the SAL and VPA mice injected with vehicle or AQ. AQ injection in SAL mice significantly increased GAD67 expression (*p* = 0.021) (Fig. 5G). D2 and D1 expression levels and the relative expression level of D2 compared to D1 (*p* = 0.095) were not significantly different (Fig. 5H–J).

### Nurr1 expression is increased in the striatum of PatDp^+/−^ mice, a genetic animal model of ASD

To investigate the importance of Nurr1 in the pathophysiology of ASD, we assessed Nurr1 expression in a commonly used ASD genetic mouse model, PatDp^+/-^ mice which carry a 6.3 Mb paternal duplication homologous to the human 15q11-q13 locus. Chromosomal abnormalities in this region are known to cause ASD, Prader-Willi syndrome, and Angelman syndrome in humans [34]. The duplication of this region is the most common cytogenetic abnormality leading to ASD. Notably, the Nurr1 expression level was also increased in striatal tissues from PatDp^+/−^ mice (*p* = 0.008) (Supplementary Fig. 6).

## Discussion

An MRI study of high-functioning ASD subjects 6–25 years of age revealed that caudate volume increased with development [35]. The growth rate of striatal structures for individuals with ASD increased compared to control subjects in longitudinal MRI research. This effect was specific to the caudate nucleus and correlated with the insistence on the same cluster of repetitive behaviors at preschool age [36]. Striatal functional connectivity is also aberrant in ASD patients. A resting-state positron emission tomography study detected weaker correlations in glucose consumption between the frontal cortical regions and the striatum in young adults with ASD [37]. However, resting-state functional MRI demonstrated that the functional connectivity between the striatum and the associative and limbic cortex increased in children with ASD [38]. These results suggest that the connectivity between the striatum and other brain regions differs in ASD patients and is related to the autistic behavioral phenotype. Glutamatergic innervation from the neocortex and thalamus modulates dendritic morphology in medium spiny neurons [39]. Cortical and thalamic glutamatergic inputs also potentiate the output of neurotransmission of striatal GABAergic neurons [40]. Corticostriatal synapses in striosomal neurons were reduced in the striatum of prenatally VPA-exposed mice [41]. Mice with autism-linked mutations in the DAT, which is a presynaptic transporter for DA reuptake from the synaptic cleft located at the membranes of dopaminergic nerve terminals, exhibited repetitive behaviors and deficits in social interaction [42]. Based on these reports, we examined synaptic alterations in the striatum and input signals to the striatum. Dendritic spine density was decreased in the prenatally VPA-exposed striatum, and this effect was specific to the mature type of spines (Fig. 1A–D). In addition, it was found that DMS from VPA mice showed significantly reduced neuronal activity (Fig. 1E–G and Supplementary Fig. 1A, B) while DLS did not (Fig. 1H), consistent with the results shown in Fig. 1A–D. In addition, glutamatergic and dopaminergic inputs to the striatum were decreased in prenatally VPA-exposed mice (Fig. 1K and Supplementary Fig. 1C, D).

Alterations in dorsal striatum input were reported to be involved in promoting sociability deficits and repetitive behaviors [8]. Human functional MRI experiments showed that the striatum became active in relation to others’ reward situations and during social learning [5]. Our behavioral studies showed a developmental delay (Supplementary Fig. 7A, G, H), an impairment of social interaction (Supplementary Fig. 7B, C), a tendency of increased grooming (Supplementary Fig. 7D), a significantly increased repetitive motor routine learning rate (Supplementary Fig. 7E), and significantly decreased motor function (Supplementary Fig. 7F). Taken together, our findings indicate that altered synaptic plasticity and glutamatergic and dopaminergic inputs in the striatum modulate striatal neurotransmission output. Thus, prenatal exposure to VPA appears to profoundly disrupt the neural circuitry in the striatum required for development, social interaction, and repetitive routine learning.

Our RNA-seq analyses identified the upregulation of synaptic and neuronal function-related genes and the downregulation of genes related to the binding and processing of proteins and bone development. Validation of the top 10 differentially upregulated genes using qPCR revealed the upregulation of only two genes. This result was found because we created the list of differentially upregulated genes following the order of fold change, not *q*-value (Fig. 2A–G). Prenatally VPA-exposed mice showed increased Nurr1 mRNA and protein levels in the striatum (Fig. 2E, H). Nurr1 (*Nr4a2*), Nur77 (*Nr4a1, NGFI-B*), and Nor-1 (*Nr4a3*) are orphan nuclear receptors and conform to the Nur subfamily. Nurr1 is expressed exclusively in brain tissue, unlike Nur77 and Nor-1. Several lines of evidence indicated that Nurr1 was important in the development and differentiation of dopaminergic neurons, neurogenesis, and learning and memory. Nurr1 is first expressed at E10.5 in mice [43] and interacts with Pitx-3 to induce the differentiation of dopaminergic precursor cells to tyrosine hydroxylase-positive dopaminergic neurons [44].

Several studies have revealed the dysregulation of Nurr1 in ASD. Comparisons of genome and exome sequencing data from ASD families revealed a frameshift variant in Nurr1 [29]. Several de novo deletions covering Nurr1 were reported in patients with ASD and intellectual disability [45, 46]. A meta-analysis of *de novo* variants in 4773 published ASD trios and 465 SPARK trios revealed a *de novo* damaging variant in the Nurr1 gene [47]. Nurr1 is also reported to be involved in reward-seeking behavior [48]. Dopaminergic neurons are the basis of the reward system, and their dysregulation is related to the disruption of circadian rhythm [49]. A growing body of research has identified significant sleep problems, low levels of melatonin, and circadian rhythm changes in children with autism [50–52]. In addition, children with increased sleep problems tend to have more behavior problems [53, 54]. There is evidence that Nurr1 regulates circadian gene expression. Nurr1 competes with the circadian nuclear receptor REV-ERBα for the regulation of circadian TH expression via a target-dependent antagonistic mechanism. In addition, Nurr1 regulates vasoactive intestinal protein gene expression, and loss of Nurr1 function results in a decrease in vasoactive intestinal protein mRNA levels within the developing midbrain [55, 56]. Therefore, our findings support the hypothesis that Nurr1 dysregulation plays a role in ASD and is related to the pathogenesis of ASD.

To illustrate the role of Nurr1 in social behavior, we performed intrastriatal injections of Nurr1-shRNA-expressing lentiviruses. As depicted in Fig. 3, VPA mice injected with sh-Nurr1 lentiviruses showed reduced expression of Nurr1 in striatal neurons and rescued social interaction. We confirmed that VPA mice injected with sh-Nurr1 lentiviruses exhibited increased total and mature spine density in the DMS (Fig. 4A–C). The expression levels of DAT and D1 were increased in the striatum of VPA mice injected with sh-Nurr1 lentiviruses. We also found that the relative expression level of D2 compared to D1 was significantly decreased (Fig. 4E–H). Medium spiny neurons are composed of D1- and D2-expressing GABAergic neurons. D1s (excitatory) are predominantly expressed on GABAergic medium spiny neurons in the dorsal striatum as part of the “direct pathway” to the globus pallidus interna (GPi) and substantia nigra pars reticularis (SNpr). D2s (inhibitory) are predominantly expressed on medium spiny neurons that primarily project to the globus pallidus externa (GPe). A recent study of postmortem brains of ASD patients reported significant increases in D2 mRNA within medium spiny neurons in the caudate and putamen, which correlates with our results using lentivirus injection shown in Fig. 1I. These results indicate the alterations in the indirect pathway of the basal ganglia, with possible implications for the E/I balance in the direct/indirect feedback pathways via thalamic and motor cortical areas [57].

To further investigate the effect of Nurr1 activation in striatal function and social behavior, we injected the Nurr1 agonist AQ in SAL mice. AQ is an anti-malaria drug that stimulates the transcriptional function of Nurr1 via physical interaction with its ligand-binding domain [58]. Several recent studies reported that the pharmacological stimulation of Nurr1 using AQ improved cognitive function via enhancement of hippocampal neurogenesis [59, 60]. Notably, AQ-injected SAL mice exhibited dysfunctions in social interaction, but the injection did not significantly affect repetitive behavior or motor function. Activity in human striatal circuits correlates with social deficits relevant to autism [7, 61]. AQ-injected SAL mice showed decreased dendritic spine density in the DMS in our results, and this effect was specific to the mature type of spines. The administration of AQ increased the expression of GAD67, which is a GABAergic neuron marker (Fig. 5G). Striatal D2 overexpression leads to a deficit in inhibitory transmission and dopamine sensitivity [62], which suggests that the molecular changes in the striatum of AQ-injected SAL mice contribute to the prefrontal cortex GABAergic system hypofunction and reduced social novelty. Together with our finding of increased Nurr1 expression and decreased mature spine density in the striatum of VPA mice, the results from VPA mice with Nurr1 knockdown and AQ-injected mice suggest the correlation between the expression of Nurr1 in the striatum and the social preference deficits in ASD.

Our study is the first study to show that Nurr1 is involved in the pathogenesis of ASD and indicates that the upregulation of Nurr1 expression in the striatum of VPA mice is the cause of the altered dendritic spine density of mature forms and may contribute to autistic behavior. These findings support Nurr1 as a therapeutic target for ASD.

## Supplementary information

Supplementary materials
