# Supplementary material for: Transcriptomic analysis in the striatum reveals the involvement of Nurr1 in the social behavior of prenatally valproic acid-exposed male mice
Source: Transl Psychiatry. 2022 Aug 9;12:324. doi: 10.1038/s41398-022-02056-z (PMC9363495; doi:10.1038/s41398-022-02056-z)
Supplement: Supplementary file 1 — Supplementary materials [file 41398_2022_2056_MOESM1_ESM.doc]

**[Supplementary Materials]**

**Supplementary Figures**


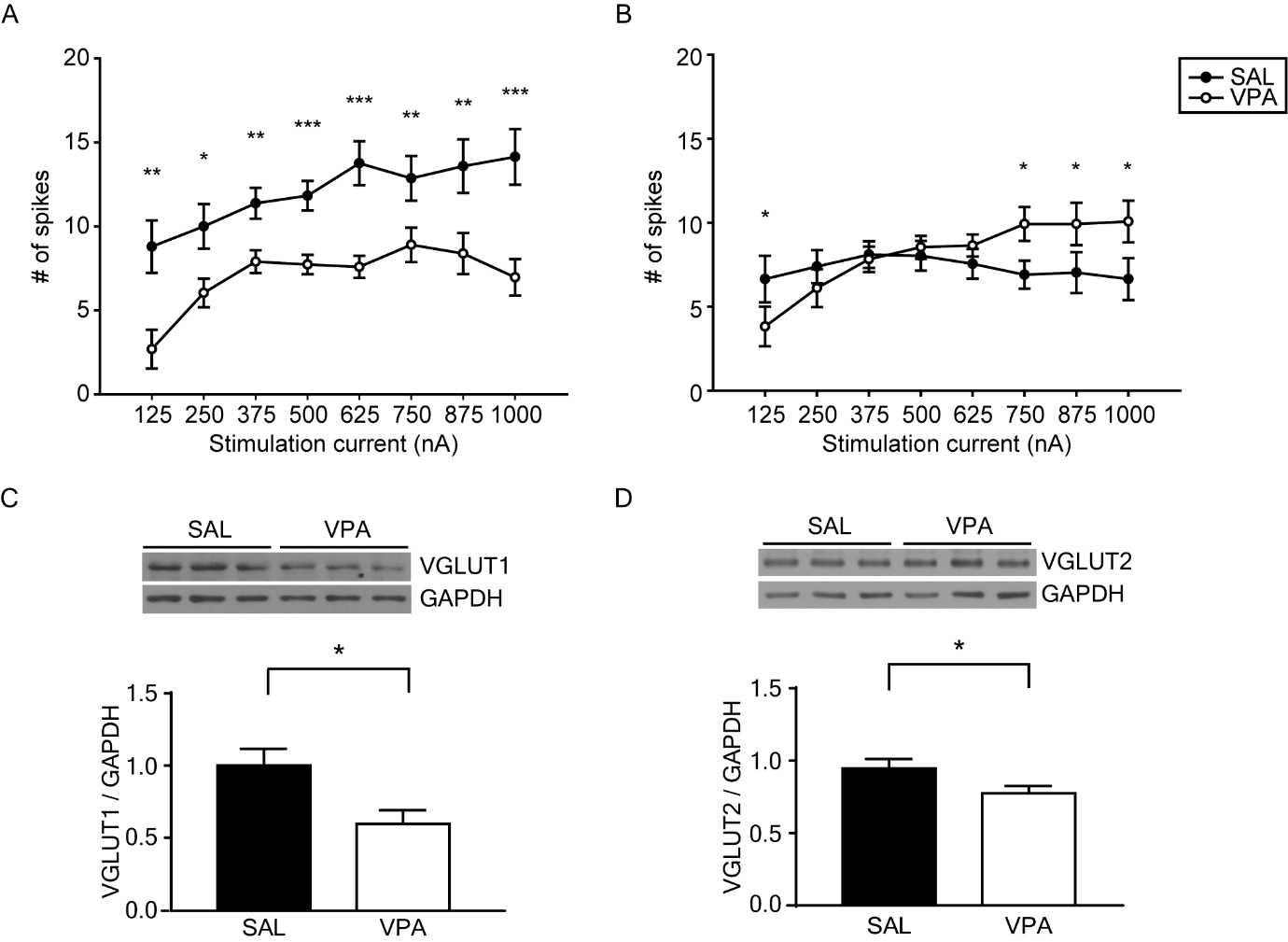


**Supplementary Fig. 1**. Abnormalities in neuronal activities and synaptic inputs in the striatum of prenatally VPA-exposed 10-week-old mice.

Line plot showing the number of stimulated firings of the striatal neurons in (A) DMS (125 nA, *p* = 0.002; 250 nA, *p* = 0.028; 375 nA, *p* = 0.001; 500 nA, *p* = 0.0003; 625 nA, *p* = 0.0003; 750 nA, *p* = 0.005; 875 nA, *p* = 0.008; 1000 nA, *p* = 0.001, SAL, n = 20, VPA, n = 16 brain slices from 6 mice, respectively) and (B) DLS (125 nA, *p* = 0.026; 250 nA, *p* = 0.159; 375 nA, *p* = 0.873; 500 nA, *p* = 0.507; 625 nA, *p* = 0.283; 750 nA, *p* = 0.027; 875 nA, *p* = 0.040; 1000 nA, *p* = 0.018, SAL, n = 14, VPA, n = 14 brain slices from 6 mice, respectively) after each stimulation current. **p* < 0.05, ***p* < 0.01, ****p* < 0.001 compared to SAL mice, Mann-Whitney test. (**C-D)** Densitometric analysis of striatal VGLUT1 levels (SAL, n = 8; VPA, n = 8), and VGLUT2 levels (SAL, n = 20; VPA, n = 24). Data are presented as the means ± SEM. **p* < 0.05 compared to SAL mice, unpaired *t*-test.


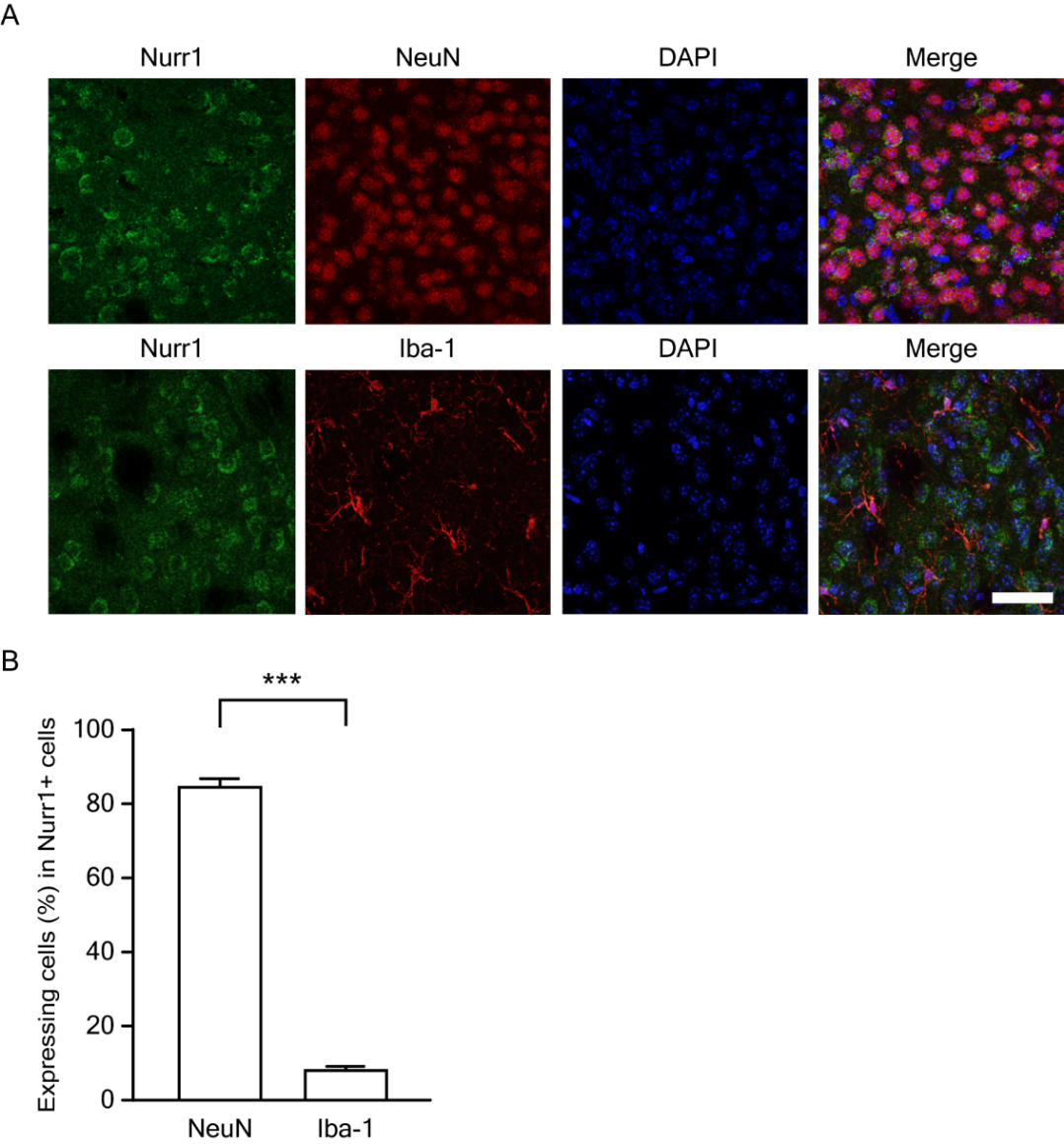


C

**Supplementary Fig. 2**. Nurr1 expression in each cell type of the striatum.

(A) Representative images of Nurr1 expression in neurons and microglia of the striatum. (B) Nurr1 is mostly expressed in neurons, not in microglia (NeuN, n = 12; Iba-1, n = 6). n means a number of mice analyzed. Scale bars: 20 μm. ****p* < 0.001 compared to percent of Iba-1 expressing cells in Nurr1 positive cells, unpaired t-test.


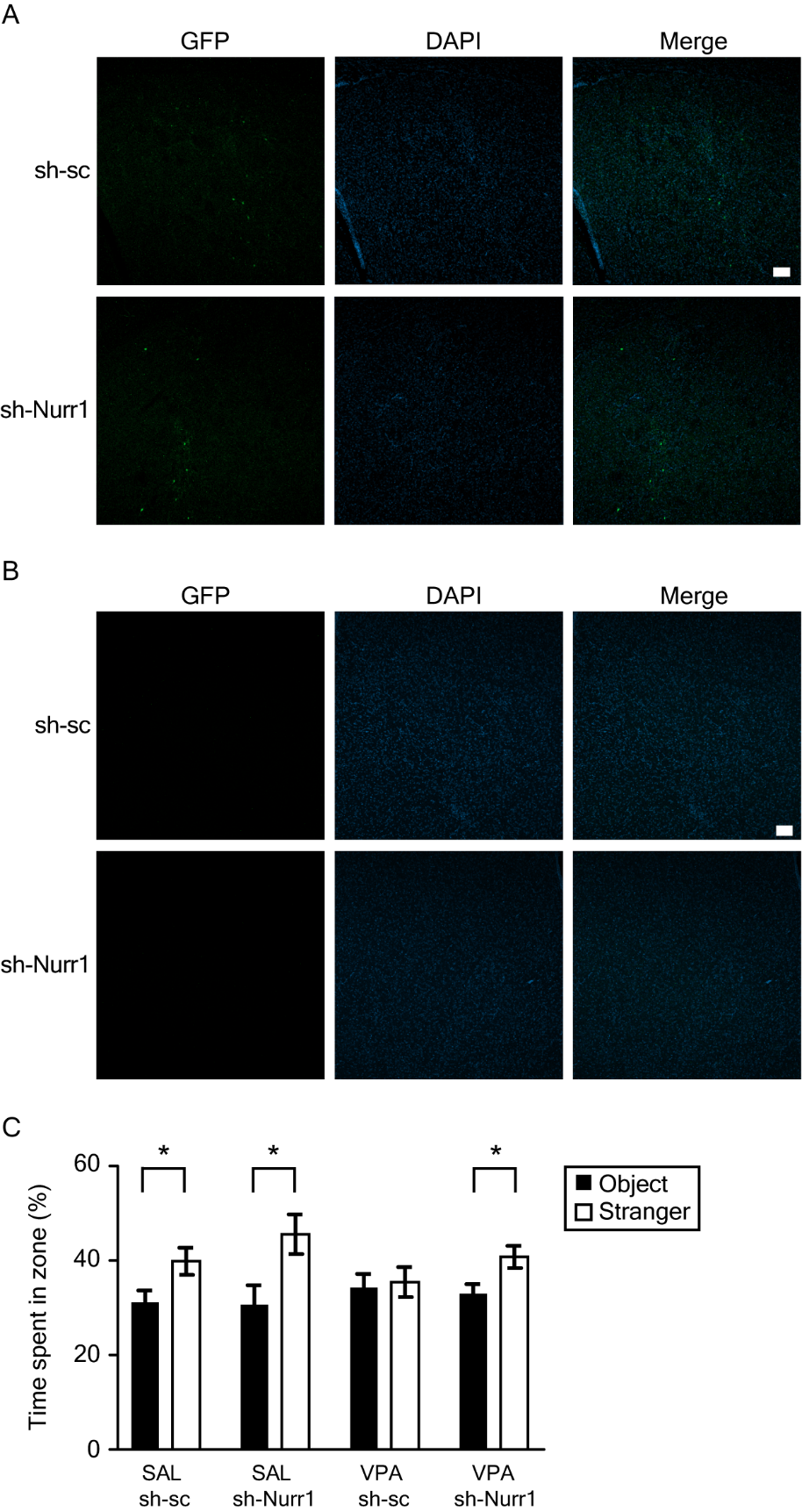


**Supplementary Fig. 3**. Lentiviral Nurr1 knockdown in the striatum rescued social deficits in prenatally VPA-exposed mice.

Representative images of GFP for lentiviral spread and DAPI for nucleus staining in dorsal striatum (A) and ventral striatum (B). Scale bars: 20 μm. (C) The interaction time with familiar and novel mice (F(3, 60) = 0.5049, *p* = 0.6802, interaction; F(3, 60) = 0.343, *p* = 0.7943, group; F(1, 60) = 23.34, *p* < 0.001, familiar vs. novel, two-way ANOVA, SAL sh-sc, n = 8; SAL sh-Nurr1, n = 8; VPA sh-sc, n = 9; VPA sh-Nurr1, n = 9). **p* < 0.05 compared to time in zone with familiar mouse, unpaired t-test.


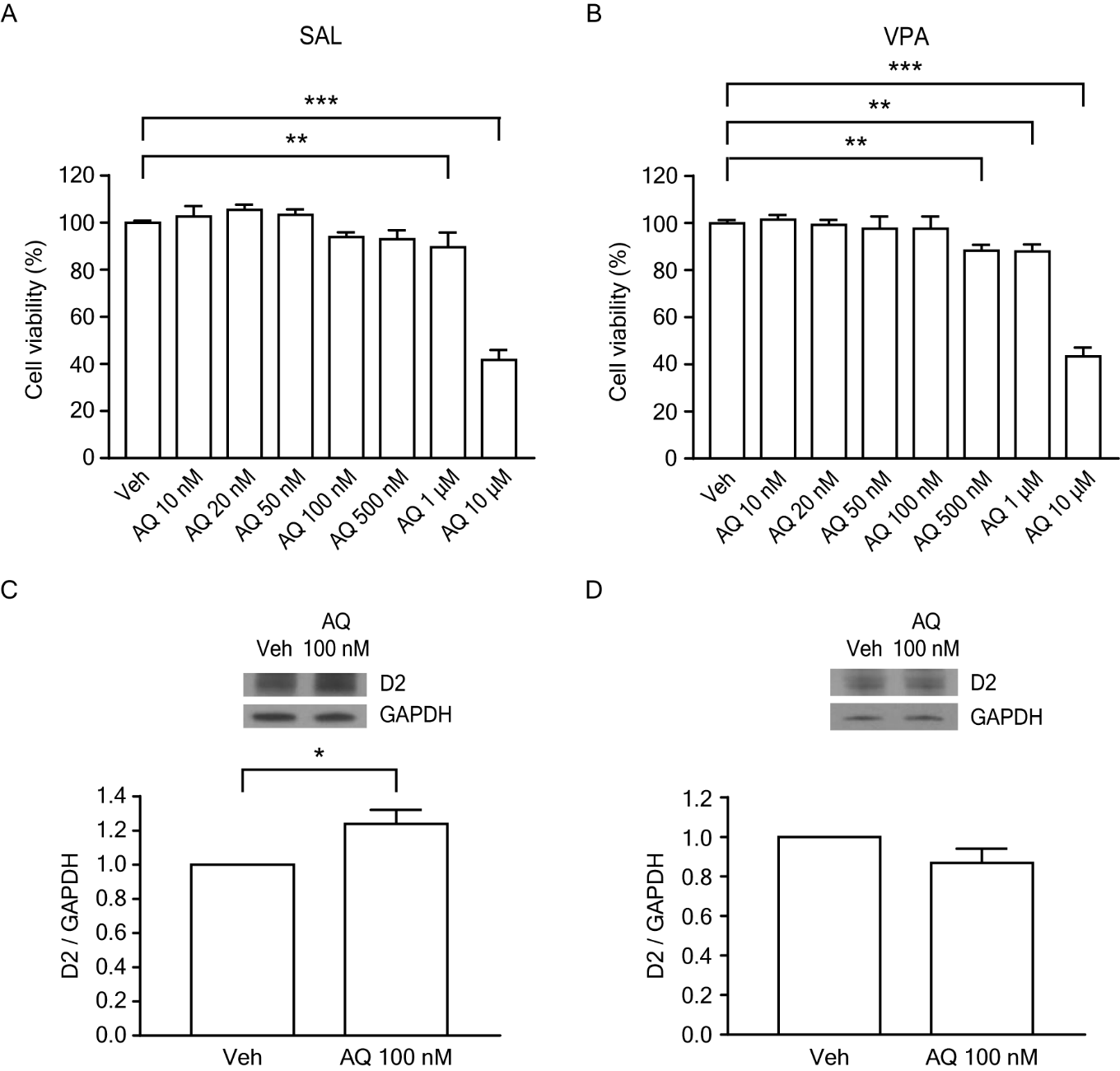


**Supplementary Fig. 4.** Increase of D2 expression in the primary striatal neuron culture from saline-exposed mice after Nurr1 activation by AQ treatment.

(A-B) Neuronal viabilities assessed with MTT assay at 24 h after the addition of AQ in primary striatal neuron culture from SAL mice, and from VPA mice (n = 6-20). n means a number of replicates analyzed. ***p* < 0.01, ****p* < 0.001 compared to Veh, 1-way ANOVA. (C-D) D2 expression at 24 h after the addition of 100 nM of AQ in primary striatal neuron culture from SAL mice, and from VPA mice (SAL Veh, n = 4; SAL AQ, n = 5, VPA Veh, n = 4; VPA AQ, n = 5). **p* < 0.05 compared to Veh, unpaired t-test.


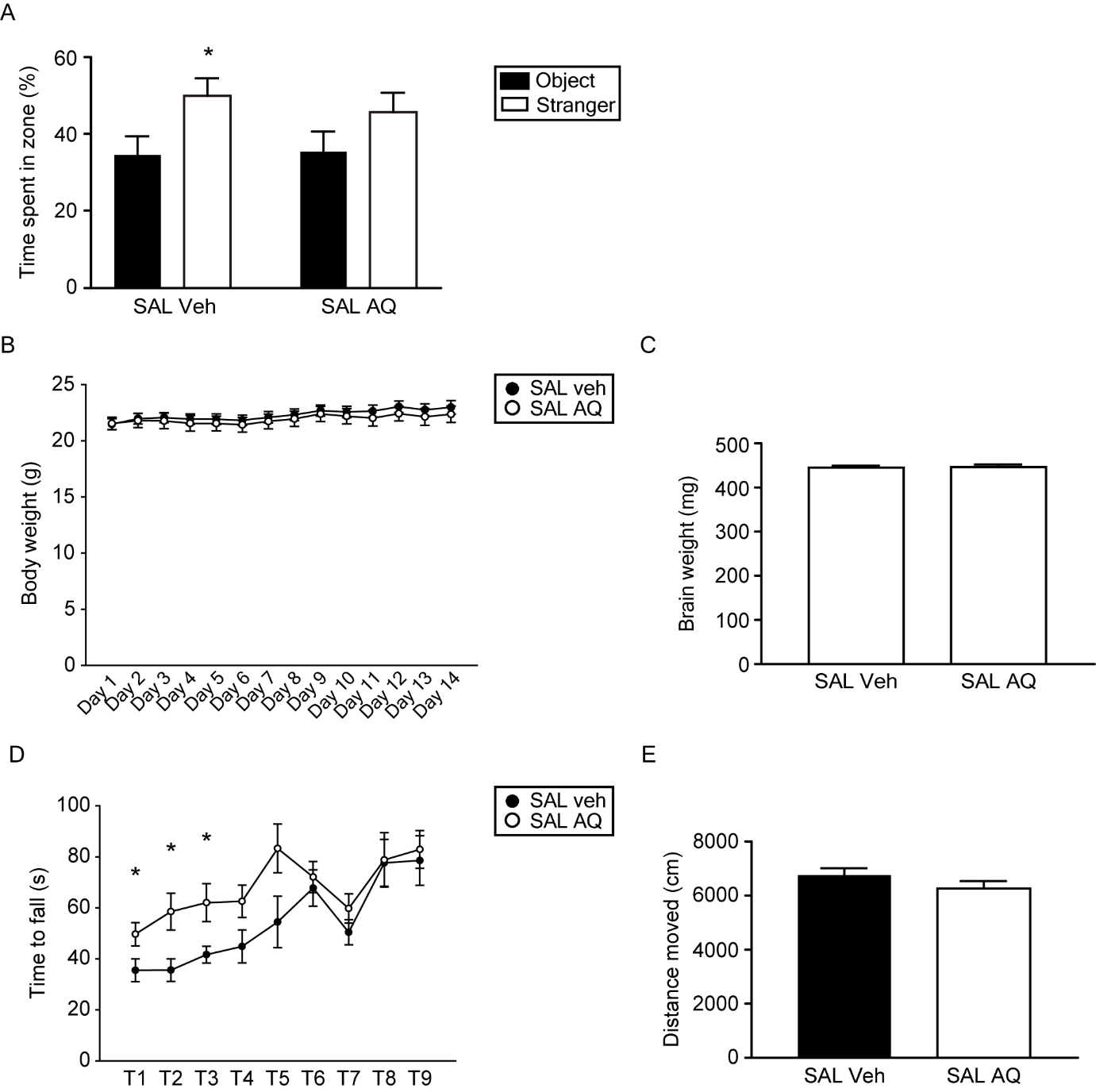


**Supplementary Fig. 5**. Administration of AQ increases the acquisition of motor routines in 10-week-old mice.

(A) The interaction time with familiar and novel mice (F(1, 40) = 0.2686, p = 0.6071, interaction; F(1, 40) = 0.1163, p = 0.7348, group; F(1, 40) = 6.766, p = 0.0130, object vs. stranger, two-way ANOVA, SAL Veh, n = 11; SAL AQ, n = 11). **p* < 0.05 compared to time in zone with object, unpaired *t*-test. (B-C) Body weight and brain weight (SAL Veh, n = 16; SAL AQ, n = 14). (D) The acquisition rates of repetitive motor routines during rotarod training (SAL Veh, n = 10; SAL AQ, n = 10). (E) The distance moved in the open field test (SAL Veh, n = 12; SAL AQ, n = 11). n means a number of mice analyzed. **p* < 0.05 compared to SAL Veh mice, unpaired t-test.


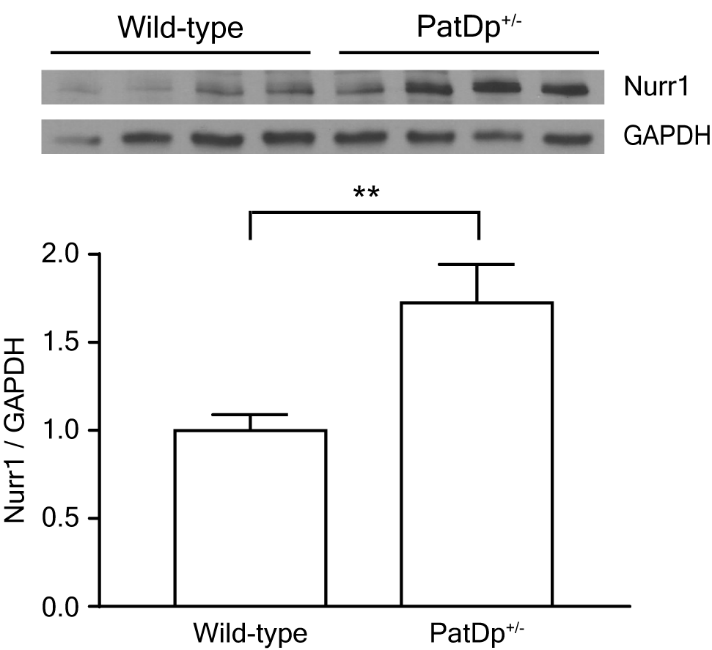


**Supplementary Fig. 6**. Nurr1 expression was also increased in another model of ASD, PatDp^+/-^ mice.

Nurr1 expression level was found to be increased in the striatum of PatDp^+/-^ mice compared to that of wild-type mice. (Wild-type, n = 11; PatDp^+/-^, n = 12). n means a number of mice analyzed. ***p* < 0.01 compared to wild-type mice, unpaired t-test.


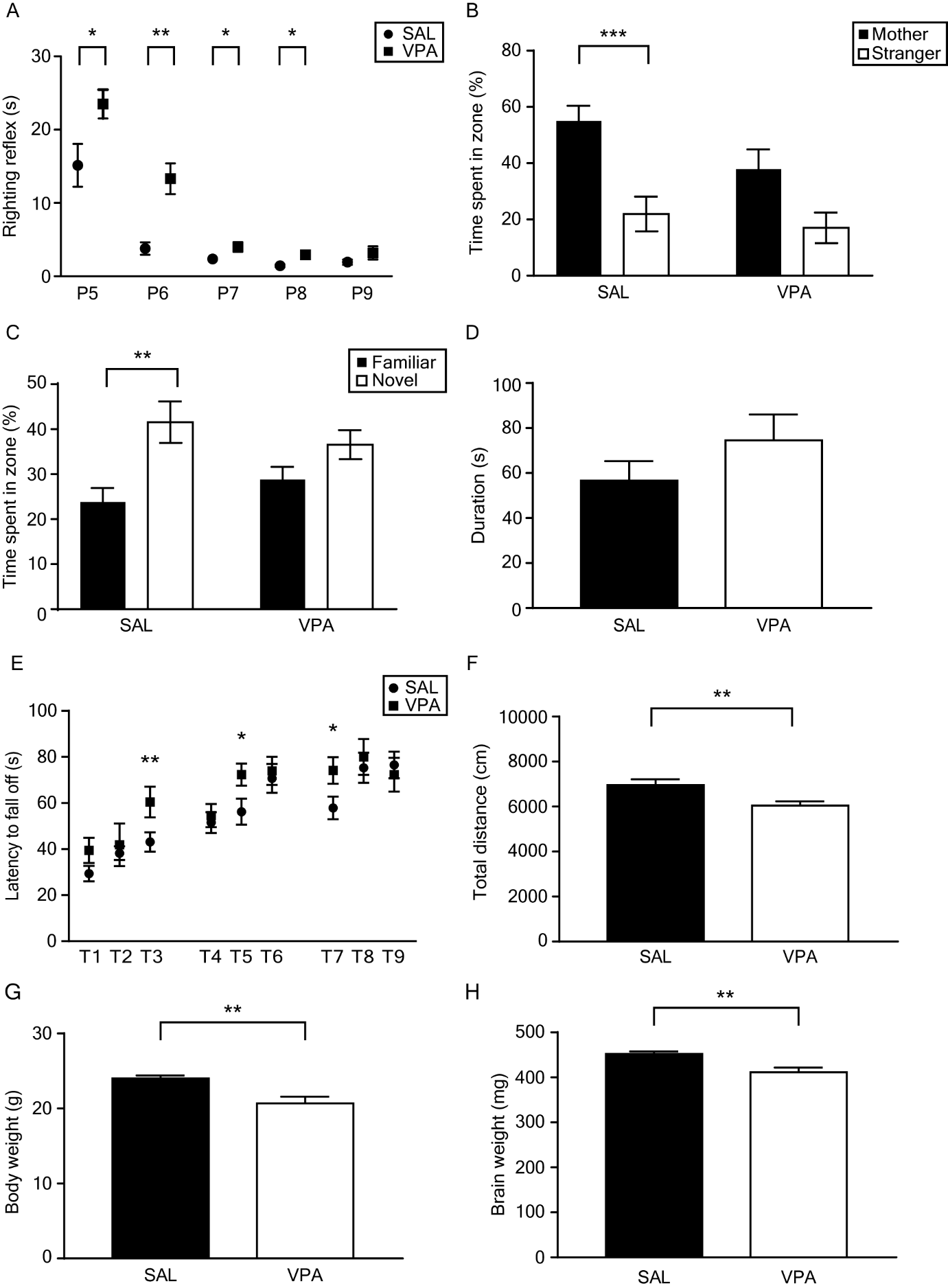


**Supplementary Fig. 7.** Deficits in social interaction, repetitive motor learning, motor function, and development of the prenatally VPA-exposed mice.

(A) The time spent until self-righting (SAL, n = 7; VPA, n = 9). **p* < 0.05, ***p* < 0.01

compared to SAL mice. (B) The time spent in mother-scented zone and in stranger-scented

zone (SAL, n = 9; VPA, n = 9). ****p* < 0.001 compared to mother-scented zone. (C) The

interaction time with familiar and novel mouse (SAL, n = 11; VPA, n = 10). ***p* < 0.01

compared to zone with familiar mouse. (D) The duration of stereotypical self-grooming

(SAL, n = 9; VPA, n = 10). (E) The acquisition rates of repetitive motor routines during

rotarod training (SAL, n = 19; VPA, n = 12). **p* < 0.05, ***p* < 0.01 compared to SAL mice.

(F) The distance travelled in the open field test (SAL, n = 19; VPA, n = 11). ***p* < 0.01

compared to SAL mice. (G-H) The body weight and brain weight (SAL, n = 9; VPA, n = 6).

***p* < 0.01 compared to SAL mice. n means a number of mice analyzed, unpaired t-test or

Mann-Whitney test.


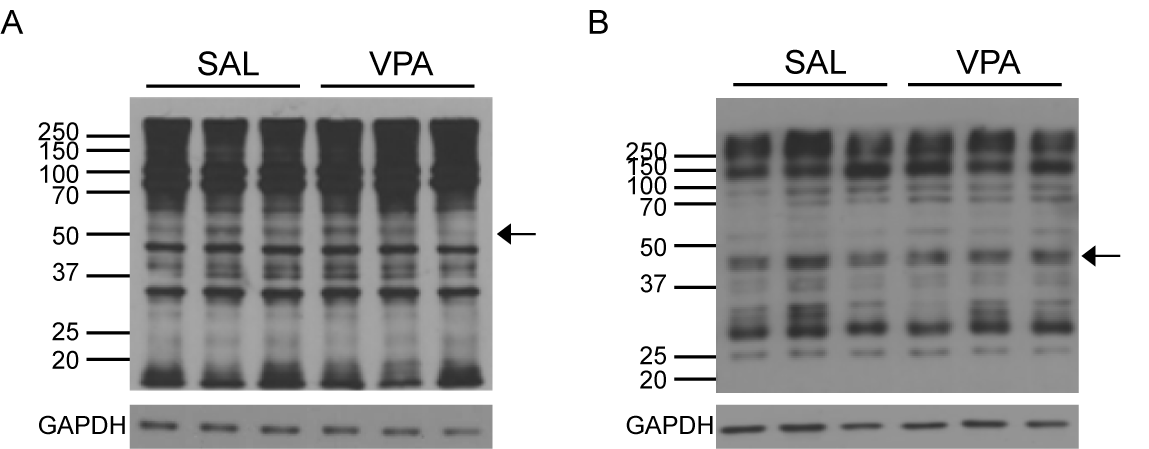


**Supplementary Fig. 8.** Representative images of D2 and D1 receptor expression levels in the striatum of prenatally VPA-exposed mice.

Full blot images of striatal D2 levels (A) and D1 levels (B) in the striatum are shown.

**Supplementary Methods**

***Behavioral assays***

All behavior tests were performed between 12:00 pm–6:00 pm. The analysis was performed by an operator blinded to the groups.

The self-righting test was held on postnatal days 5-9 (P5-9) as described in previous literature. Each mouse was placed in the supine position and gently held with all four limbs extended outwards at which time it was released. The time taken to the righting was recorded by the latency for all four paws touching the surface. A maximum score of 30 sec was recorded when the mouse failed to right in that period.

The maternal scent preference test was conducted on P14 as described in previous research ^21^. Each pup was moved from the home cage to a fresh transparent polycarbonate cage (20 cm × 30 cm × 15 cm). The left third of the test cage was filled to a depth of 3 cm with litter from the mother’s cage, the center third contained clean litter, and the right third contained litter from the cage of a stranger dam. The placement of the test litters (mother and stranger) was alternated across subjects to control for any side preferences. Three 1-min trials, with intertrial intervals of 10 sec, were administered for each pup. For the first trial, pups were placed in the center of the fresh litter facing the back wall of the test cage. For the second trial, the pup was placed in the center of the fresh litter facing the section containing its mother’s cage litter. For the third trial, the pups faced the section containing the litter of the stranger dam. The time spent in each section of the cage was recorded and averaged across the 3 trials.

The self-grooming test was performed at 9-10 weeks of age, and each mouse was placed individually into a clean transparent polycarbonate cage (20 cm× 30 cm × 15 cm) with a video camera placed 15 cm away from the cage. The duration of the test was 10 min after 10 min habituation. The time spent grooming was measured.

The rotarod test was conducted with 9 to 10-week-old mice following previously published research ^15^. The test consisted of three trials per day over the course of 3 days. Rotarods were accelerated from 4–40 rpm in 300 s. Each trial ended when a mouse fell off, made one complete backward revolution while hanging on, or reached 300 s.

The open field test was conducted at 9 to10 weeks of age. A square plastic box (100 cm × 100 cm × 40 cm) was used for this general locomotor activity test. The mice were put into the arena and its movements monitored with a video camera for 30 min. Tracking of mouse behavior was done using EthoVision XT (Noldus) tracking system.

***Western blot***

Antibodies used were Nurr1 (#PA5-13416, Thermo Fisher Scientific, IL, USA), Cbln1 (#ab-64184, Abcam, Cambridge, UK), D2 (#AB5084P, Millipore, CA, USA), D1 (#ab20066, Abcam, Cambridge, UK), Vesicular glutamate transporter 1 (VGLUT1, #48-2400, Invitrogen, CA, USA), Vesicular glutamate transporter 2 (VGLUT2, #135 403, Synaptic systems), Dopamine transporter (DAT, #MAB369, Millipore, CA, USA), Glutamate decarboxylase 67 (GAD67, #ab26116, Abcam, Cambridge, UK). Relative intensity of blots was quantified using ImageJ software.

***AQ treatment***

For an in vitro study, primary striatal neuron cultures were treated with 100 nM of amodiaquine (AQ, Sigma-Aldrich, MO, USA) for 24 h. For in vivo study, mice were intraperitoneally injected with AQ (20 mg/kg), twice per day at 12 h intervals, for 2 weeks. Mice underwent behavioral testing 1 week after the final injection.

***Immunofluorescence***

The primary antibodies used were Nurr1 (#PA5-13416, Thermo Fisher Scientific, IL, USA), NeuN (#MAB377, Millipore, CA, USA), Iba-1 (#NB100-1028, Novusbio, CO, USA). Secondary antibodies used were goat anti-rabbit Alexa 555, goat anti-mouse Alexa 488, goat anti-rabbit Alexa 488, and donkey anti-goat Alexa 555 (Thermo Fisher Scientific, IL, USA).

***PatDp/+ transgenic mice***

PatDp/+ transgenic mice were previously created in the Takumi Lab. PatDp/+ mouse has an interstitial 6.3 Mb duplication on chromosome 7 which corresponds to human 15q11-q13. Striatal tissues of PatDp/+ mice were generous gift from Dr. Jong-Cheol Rah (Korea Brain Research Institute).
